# Intraoperative Complications of Cataract Surgery Using Intracameral Illumination in the Elderly over 75 Years

**DOI:** 10.1155/2019/1594152

**Published:** 2019-01-10

**Authors:** Yu Jeong Kim, Su Jin Park, Jong Yeon Lee, Dae Yeong Lee, Dong Heun Nam

**Affiliations:** Department of Ophthalmology, Gachon University Gil Hospital, Incheon, Republic of Korea

## Abstract

**Purpose:**

To evaluate intraoperative complications and utilization of adjunctive devices between microscope and intracameral illuminations during cataract surgery in the elderly over 75 years.

**Design:**

A retrospective, consecutive, interventional case series *Participants.* Two hundred eighty-six eyes of 184 patients older than 75 years who underwent cataract surgery using microscope and intracameral illuminations.

**Methods:**

A chart review was performed on an advanced cataract surgery group of 141 consecutive cases in which the intracameral illumination was used and on a standard cataract surgery group of 145 consecutive cases in which the intracameral illumination was not used.

**Main Outcome Measures:**

Intraoperative complications (posterior capsule rupture, radial tear of the anterior capsule, dropped nucleus, or sulcus-implanted/sclera-fixated IOL) and utilization of adjunctive devices (pupil expansion device or anterior capsule staining).

**Results:**

The frequency of use of the pupil expansion device was lower in the advanced cataract surgery group than that in the standard cataract surgery group (0.7% vs 6.9%; *p*=0.007). Furthermore, the rates of a posterior capsule rupture and at least one intraoperative complication were lower in the advanced cataract surgery group than those in the standard cataract surgery group (0.7% vs 4.8%; *p*=0.067) (0.7% vs 7.6%; *p*=0.004).

**Conclusions:**

In the current cohort of patients over 75 years, the rate of intraoperative complications was lower when using the intracameral illumination than that when using the conventional method. Cataract surgery using intracameral illumination would be good option for elderly people.

## 1. Introduction

Modern cataract extraction generally provides excellent visual outcomes. Good visualization of the lens structures is essential for safe and effective performance of phacoemulsification cataract surgery. Although reported to occur in only 1% to 5% of procedures, posterior capsule rupture with or without vitreous loss is a dreaded complication of cataract surgery that significantly impacts the patient's postoperative visual outcome and cost of cataract surgery [1]. Numerous clinical entities have been identified as risk factors; most are age dependent, with advanced age being a significant risk factor for posterior capsule rupture [1–3]. Older age may contribute to posterior capsule rupture due to weakening of the zonules with advancing age, increased lens density, and other age-related ocular or systemic comorbidities that elevate the complexity of the cataract surgery performed [2–4].

The effect of age may be explained by coexisting conditions such as small pupil, pseudoexfoliation, glaucoma, and advanced cataract [5, 6]. Utilization of adjunctive tools such as pupil expansion or capsule staining in the setting of challenging cataract cases can significantly limit adverse intraoperative outcomes and result in reproducible surgical success. However, their increased cost, increased time requirement, iris trauma, or inadvertent posterior capsule staining may limit their widespread use [7–10].

An advanced cataract surgery technique using the intracameral illumination has been introduced with an enhanced 3D effect and an improved depth perception of lens. Increasing clinical experience with the intracameral illumination has provided us with advantages increasing the safety of cataract surgery. Even without anterior capsule staining or pupil expansion device, the advanced technique simplified the challenging cataract surgery [11–16].

In a recent study, patients aged 75 years and over were at least 37% more likely to have complex cataract surgery than patients aged 65 to 69 years [17]. The objective of this study was to compare intraoperative complications and utilization of adjunctive devices between microscope and intracameral illuminations during phacoemulsification cataract surgery in the elderly over 75 years.

## 2. Methods

Data were analyzed in a retrospective, consecutive, interventional case series of 286 cataract surgeries from January 2013 to December 2016. This study was exempted from Gachon University IRB (GBIRB 2017-345), and the procedures used conformed to the tenets of the Declaration of Helsinki. Inclusion criteria were patient age greater than 75 years and surgical indication of senile cataracts. Exclusion criteria were eyes with prior trauma or previous intraocular surgery. All operations were performed by 3 experienced surgeons.

A chart review was performed on an advanced cataract surgery group of 141 consecutive cases in which the intracameral illumination was used and on a standard cataract surgery group of 145 consecutive cases in which the intracameral illumination was not used. The advanced cataract surgery using intracameral illumination described in our previous studies [11–15] was used in the treatment group. A review of medical records was performed in which demographic data, medical history, ocular history, best-corrected visual acuity, slit-lamp examination including lens scoring (Lens Opacities Classification System III [18]), dilated fundus examination, and fundus photography were collected and reviewed.

For assessing the risk of complications in phacoemulsification cataract surgery, the risk score was calculated for each case using the Buckinghamshire risk stratification system [19]. Main outcome measures were intraoperative complications (posterior capsule rupture, radial tear of the anterior capsule, dropped nucleus, or sulcus-implanted/sclera-fixated IOL) and utilization of adjunctive devices (pupil expansion device or anterior capsule staining).

Student's *t*-test, chi-square test, and Fisher's exact test were used to test significance of differences between the advanced and standard cataract surgery groups. *p* values of 0.05 or less were considered statistically significant.

## 3. Results

Demographic characteristics were similar across all cases with no clinically significant differences between the advanced and standard cataract surgery groups. The mean age of the patients was 79.41 years (SD 4.03; range 75–96) in the advanced cataract surgery group and 79.72 years (SD 4.40; range 75–95) in the standard cataract surgery group (*p*=0.541). Based on the Buckinghamshire risk stratification system, however, the risk score (2.84 ± 2.19) in the advanced cataract surgery group was higher than that (1.81 ± 1.74) in the standard cataract surgery group (*p* < 0.001) (Table 1).

In the 145 standard cataract surgery cases, the surgeons chose to use the pupil expansion device 10 times (6.9%). In the 141 advanced cataract surgery cases, nonetheless, the surgeons chose to use it only once (0.7%). The frequency of use of the pupil expansion device was significantly lower in the advanced cataract surgery group than that in the standard cataract surgery group (*p*=0.007). In the 145 standard cataract surgery cases, the surgeons chose to use the anterior capsule staining 3 times (2.1%). In the 141 advanced cataract surgery cases, nevertheless, the surgeons did not use it at all (0%). The frequency of use of the anterior capsule staining was lower in the advanced cataract surgery group than that in the standard cataract surgery group but not significant (*p*=0.248) (Table 2).

In the standard cataract surgery group, a posterior capsule rupture, a radial tear of the anterior capsule, and a dropped nucleus occurred in 7 eyes (4.8%), 4 eyes (2.8%), and 3 eyes (2.1%), respectively. An IOL was implanted in the capsular bag in 134 eyes (92.4%) and in the ciliary sulcus or sclera fixated in 9 eyes (6.2%) with capsule tears. In the advanced cataract surgery group, however, a posterior capsule rupture occurred in only one eye (0.7%). Neither a radial tear of the anterior capsule nor a dropped nucleus occurred. An IOL was implanted in the capsular bag in 140 eyes (99.3%) and in the ciliary sulcus in only one eye (0.7%) with a posterior capsule rupture. At least one intraoperative complication was detected in 11 eyes (7.6%) in the standard cataract surgery group but in only one eye (0.7%) in the advanced cataract surgery group (*p*=0.004) (Table 3).

**Figure 1:**
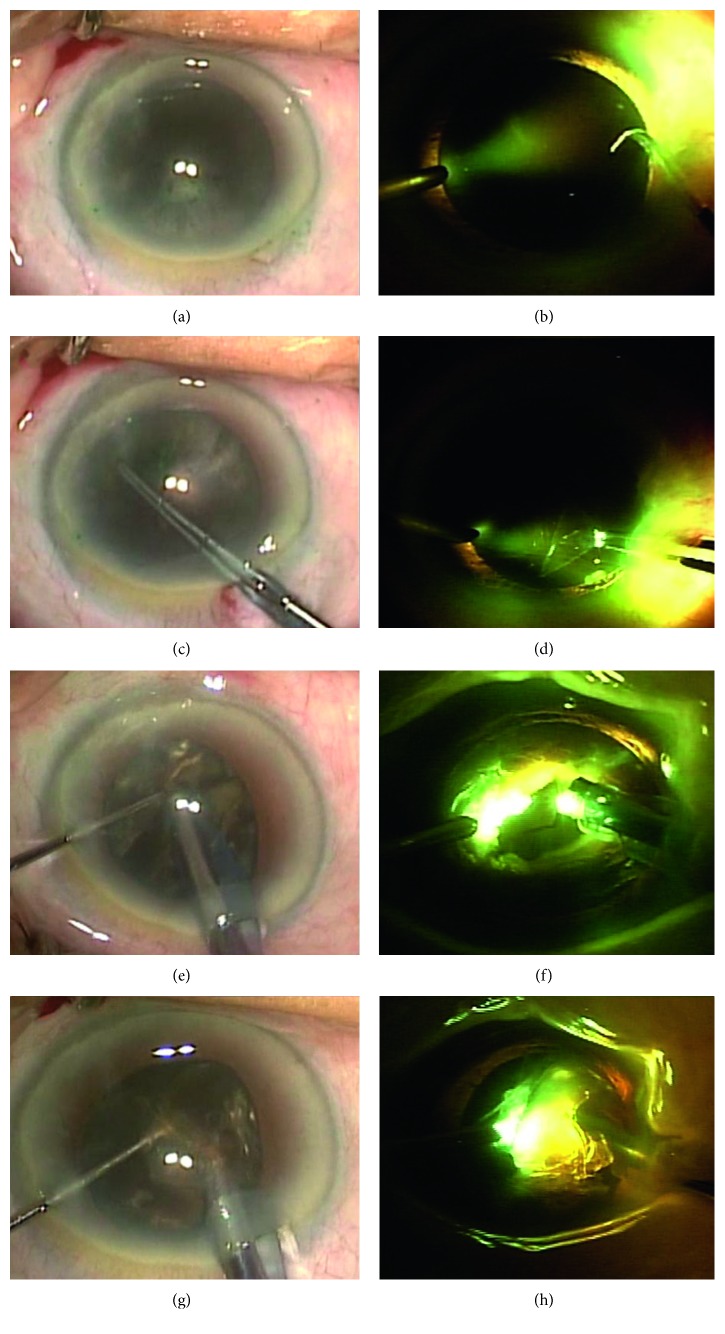
An 80-year-old female patient with brunescent cataract and severe arcus senilis who underwent cataract surgery without the intracameral illumination in the right eye (a, c, e, g) but with it in the left eye (b, d, f, h). (a) Anterior capsule staining using the indocyanine green dye. (b) Enhanced visibility of the anterior capsule with the intracameral illumination. (c) Limited visibility of the capsulorhexis. (d) Improved visibility of the capsulorhexis. (e) Incomplete nucleofractis crack. (f) Enhanced depth trench and complete nucleofractis crack. (g) Poor visibility of nucleus fragments and limited depth perception. (h) Improved visibility of nucleus fragments and enhanced depth perception of the field.

A case was an 80-year-old female patient with brunescent cataract in both eyes who underwent cataract surgeries. In the right eye, a standard cataract surgery without the intracameral illumination was performed. Because the visualization of the anterior capsule was very poor under the operating microscope, anterior capsule staining was performed using the indocyanine green dye. With limited depth perception and contours of nucleus fragments, it was very difficult to perform the capsulorhexis and nucleus fragmentation. At the end of the fragmentation, the posterior capsule rupture associated with the dropped nucleus was noticed. It was managed with vitreoretinal surgery in which scleral IOL fixation was performed. While in the left eye, an advanced cataract surgery using the intracameral illumination was performed. The intracameral illumination improved depth of the surgical field and enhanced the details of the lens structures, mainly the anterior and posterior capsules. The surgery was successfully completed without any complications (Figure 1).

## 4. Discussion

Because of the demographic shift in both developed and developing countries toward older age, the proportion of patients suffering from cataracts is expected to increase, as are age-related diseases [3, 4]. However, it is difficult to perform cataract surgery on an elderly patient owing to high prevalence of preexisting ocular and systemic diseases and the difficulty of patients' cooperation during surgery. The effect of age may be explained by miosis, iris synechias, brunescent cataract, and aging of the cornea such as opacification of Descemet's membrane and corneal guttae [3–6, 20]. Often, these signs appear in the same patient, and even in the most experienced hands and in the best operative settings, cataract surgery is difficult and more prone to complications [21]. In a previous study, a 60-year-old patient had a 45% higher risk for the intraoperative posterior capsule rupture than a 50-year-old patient, whereas a 70-year-old patient had a ∼210% higher risk for the intraoperative posterior capsule rupture than a 50-year-old patient [2].

In the elderly, poorly dilated pupil, advanced lens density, and aging of the cornea prevent the red reflex produced by the coaxial light of the microscope. To resolve this difficulty, capsule dyes and pupil expansion devices have been developed. However, these devices add time, complexity, and cost to the surgical procedure. Furthermore, both intraocularly used dyes, which can be hazardous, and mechanical iris dilation, which is traumatic to the iris, can cause postoperative inflammation in the anterior segment [7–10, 22].

Previous studies for assessing the risk of complications in phacoemulsification cataract surgery have confirmed that the risk of intraoperative complications increases with higher preoperative risk scores [19]. Even with the higher risk score in the advanced cataract surgery group, however, the rate of intraoperative complications such as posterior capsule rupture was lower compared with that in the standard cataract surgery group. Additionally, the frequencies of use of both pupil expansion device and anterior capsule staining were lower in the advanced cataract surgery group. Although all conventional dyes have been known to cause significant endothelial cell density loss, the intracameral illumination does not seem to cause significant damage to the corneal endothelial cells [23]. These results suggest that the intracameral illumination improves depth perception of the field and resolution of the lens structures and reduces the technical challenges. More importantly, it decreases the risk of intraoperative complications of challenging cataract surgery in the elderly even without utilization of adjunctive devices.

This study has some limitations such as a small number of patients, small number of surgeons, retrospective nature, and incomparable baseline characteristics. Because the surgeon's skill would be a major factor related to the rate of intraoperative complications, the differences among the 3 surgeons should have been compared. Nevertheless, these analyses were not valid due to the small sample size. Because of the retrospective design of this study, preoperative risk scores for intraoperative complications were different between the treatment and control groups. Considering that all procedures were performed by 3 attending surgeons at a university-based tertiary referral center and the rate of intraoperative complications was lower in the treatment group with the higher risk score, the preoperative risk scores might not have significantly biased our results. However, the lack of randomization is the main limitation of our study. Further studies should be prospectively designed.

In conclusion, the frequency of use of both pupil expansion device and anterior capsule staining and the rate of intraoperative complications of cataract surgery in the elderly over 75 years were significantly lower when using the intracameral illumination than those when using the conventional method. These results suggest that the advanced technique simplifies the challenging cataract surgery and increases the safety of the procedure.

## Figures and Tables

**Table 1:** Preoperative characteristics.

	Illuminator group (*n* = 141)	Nonilluminator group (*n* = 145)	*p* value
Age at surgery	79.41 ± 4.03	79.72 ± 4.40	0.541^*∗*^
Sex (male/female)	48/93	44/101	0.503†
B score	2.84 ± 2.19	1.81 ± 1.74	<0.001^*∗*^

*n* = number of cases; B score = Buckinghamshire risk stratification system score; ^*∗*^Student's *t*-test; †chi-square test.

**Table 2:** Intraoperative additional procedure.

	Illuminator group (*n* = 141)	Nonilluminator group (*n* = 145)	*p* value
Iris retractor	1 (0.7%)	10 (6.9%)	0.007^*∗*^
AC staining	0 (0.0%)	3 (2.1%)	0.248†

AC = anterior capsule; ^*∗*^chi-square test; †Fisher's exact test.

**Table 3:** Intraoperative complication.

	Illuminator group (*n* = 141)	Nonilluminator group (*n* = 145)	*p* value
PC tear	1 (0.7%)	7 (4.8%)	0.067†
AC radial tear	0 (0.0%)	4 (2.8%)	0.123†
Dropped nucleus into vitreous	0 (0.0%)	3 (2.1%)	0.248†
Remnant lens in A/C	0 (0.0%)	1 (0.7%)	1.000†
Sulcus implantation or SF	1 (0.7%)	9 (6.2%)	0.019†
Number of patients with complication (≥1)	1 (0.7%)	11 (7.6%)	0.004^*∗*^

AC = anterior capsule; PC = posterior capsule; A/C = anterior chamber; SF = scleral fixation; ^*∗*^chi-square test; †Fisher's exact test.

## Data Availability

The data in this paper are available from the corresponding author upon reasonable request.
